# The Effects of a Blend of Essential Oils in the Milk of Suckling Calves on Performance, Immune and Antioxidant Systems, and Intestinal Microbiota

**DOI:** 10.3390/ani14243555

**Published:** 2024-12-10

**Authors:** Luisa Nora, Charles Marcon, Guilherme Luiz Deolindo, Mateus Henrique Signor, Ana Luiza Muniz, Miklos Maximiliano Bajay, Priscila Marquezan Copetti, Bianca Fagan Bissacotti, Vera M. Morsch, Aleksandro Schafer da Silva

**Affiliations:** 1 Graduate Program in Animal Science, Universidade do Estado de Santa Catarina (UDESC), Chapecó 89815-630, SC, Brazil; luisa.nora22@gmail.com (L.N.); charlesmarcon3@gmail.com (C.M.); guilhermeluizd@outlook.com (G.L.D.); miklos.bajay@udesc.br (M.M.B.); 2 Department of Animal Science, Universidade do Estado de Santa Catarina (UDESC), Chapecó 89815-630, SC, Brazil; mateushenriquesignor@gmail.com (M.H.S.); analuizamusouza@gmail.com (A.L.M.); 3 Graduate Program in Toxicological Biochemistry, Universidade Federal de Santa Maria (UFSM), Santa Maia 97105-900, RS, Brazil; priscilaa_marquezan@hotmail.com (P.M.C.); bianca_fbissacotti@hotmail.com (B.F.B.); veramorsch@gmail.com (V.M.M.)

**Keywords:** cinnamaldehyde, 1,8-cineole, carvacrol, thymol, intestinal microorganisms

## Abstract

The objective of the present study was to determine whether the addition of a blend based on the essential oils of cinnamon, oregano, and eucalyptus to the liquid diets of calves would stimulate the immune system combined with anti-inflammatory action, minimize oxidative responses, and alter the intestinal microbiota, consequently enhancing animal growth. Twenty-four male Holstein calves (approximately five days old) were suckled for 60 days, underwent a weaning process, and were followed up until day 75 of the experiment. The calves were divided into control (*n* = 12) and phytobiotic (*n* = 12) groups, receiving commercial milk replacer and pelleted concentrate ad libitum. For the phytobiotic group, we added the blend to the liquid diet twice daily at 5 mL/feeding in the first 15 days and 10 mL/feeding until day 60. We detected no differences in weight gain, but animals in the phytobiotic group tended to consume less feed. Calves treated with phytobiotics showed better conversion and feed efficiency than the animals in the control group. Lower leukocyte and lymphocyte counts were observed, as was a higher cholesterol concentration. Immunoglobulin A, ceruloplasmin, and transferrin also differed between groups, with higher IgA and lower levels of acute-phase proteins (ceruloplasmin and transferrin) in calves that consumed the phytobiotic. Higher glutathione S-transferase activity was found in the serum of calves in the treatment group. The intestinal microbiota did not differ between the groups; however, the genera Acinetobacter, Pseudomonas, and Psychrobacter were the most abundant regardless of treatment. We concluded that the blend of oils based on cinnamon, oregano, and eucalyptus improved the calves’ immune and antioxidant systems, improving feed efficiency without affecting the intestinal microbiota.

## Simple Summary

The results indicated that the supply of a blend of essential oils with cinnamon, oregano, and eucalyptus provided the calves with humoral and antioxidant immune system stimulation, minimizing physiological oxidative stress and leading to better efficiency. The health improvement did not enhance the growth of calves that consumed essential oils. The assessments of the abundance and biodiversity of microbiota did not differ between animals that consumed the blend of essential oils and those that did not; however, it allowed the identification of the main genera in the feces of calves in the first weeks of age.

## 1. Introduction

Dairy production is improving internationally and Brazil is one of the largest milk producers worldwide [1]. These advances have been taking place to minimize problems and enhance production. In dairy production, several phases of animal husbandry, including calf production, are considered the most critical because calves have extremely immature and fragile immune systems [2]. Calves face several problems during the first weeks of life; however, the primary problem affecting this animal class is diarrhea, a clinical sign associated with infections caused by some pathogenic microorganisms. The diarrhea problem can be more significant when accompanied by the loss of appetite and dehydration, further weakening an animal [3]. It can die quickly, depending on the degree of infection and lack of response from the animal.

Low ambient temperatures, a high number of calves housed in the same facility, a lack of heating, inadequate ventilation, and an inadequate method of supplying a liquid diet (milk and its substitutes) increase the incidence of diarrhea and promote a higher mortality rate [4]. Understanding the intestinal microbiota is essential for understanding diarrhea; however, this microbiota is also responsible for digestion, the absorption of some nutrients, and helping establish the animal’s immune system regarding protection against pathogenic microorganisms [5,6].

The intestinal microbiota in calves begins to be established from birth quickly and is affected by several factors, including the microbiota of the mother’s vaginal canal at the time of delivery, the environment in which the animal is housed, the origin of the feed provided to the animal, whether the feed is consumable or discarded milk, and the ingestion of solid feed [7,8]. Several non-pathogenic bacteria form this microbiota and can often act as barriers to other pathogenic bacteria [9]. Some bacteria in the gastrointestinal tract are harmless while the animal is healthy; however, they can cause illness when the calf is immunosuppressed. The gastrointestinal microbiota can be modulated to establish specific microorganisms, and this modulation can be accomplished in several ways, including using essential oils [10]; they are more effective when performed at the beginning of the animal’s life. A calf at this stage has a fluctuating microbiota that depends on environmental conditions [11].

Essential oils are products extracted from several plant sources [12], which, because of their antimicrobial action [13,14], have been used as alternatives to reduce the use of antibiotics in animal nutrition [14,15]. Cinnamon, eucalyptus, and (mainly) oregano oils have been frequently studied due to their biological properties, which allow effects such as antimicrobial, antioxidant, and anti-inflammatory [16,17,18] effects, and these compounds are used for additives in animal feed. Cinnamon essential oil is extracted from *Cinnamomum* spp. [19] as a source of cinnamaldehyde, the primary bioactive component [13]. Another component extracted from essential oil is carvacrol, which derives primarily from oregano (*Origanum vulgare*) and thyme (*Thymus vulgaris*) [14]. Oregano oil also acts as a performance enhancer in ruminants as it improves feed conversion and health by modulating ruminal fermentation [20]. The essential oil of eucalyptus is extracted from the leaves, and 1,8-cineole is the primary component of interest [21,22] due to its gastroprotective and antioxidant activities; it regenerates gastric cells and increases gastric mucus [23].

Essential oils, natural antimicrobials in calf production, are increasingly promising, especially in blend form. The powerful aroma of essential oils can be a problem in feeding calves because, when added to milk, they can lead to a lack of interest in consumption; in concentrate, these oils can reduce consumption. The benefits of isolated oregano [20], cinnamon [15], and eucalyptus [24] oil for young ruminants are known; the authors of [25] shows us in their work that when oregano oil is provided to unsaieded calves from 70 days of life, there is a hand weight gain and a stimulus of the immune system, with an increase in IgA and IgM levels. However, the effect of these three essential oils when combined and given to calves during the suckling phase is not known. But research tells us that the combination of essential oils can have their effects enhanced by having synergistic actions, improving antioxidant functions for example [26]. Thus, the objective of the present study was to determine whether the addition of a blend based on the essential oils of cinnamon, oregano, and eucalyptus to the liquid diets of calves would stimulate the immune system combined with anti-inflammatory action, minimize oxidative responses, and alter the intestinal microbiota, consequently enhancing animal growth.

## 2. Materials and Methods

The study was carried out at the Experimental Farm of the Centro de Educação Superior do Oeste of the State University of Santa Catarina, in the municipality of Guatambu, South of Brazil. It lasted 75 days: 60 days of suckling, 6 days of gradual weaning, and 9 days post weaning. The Committee on Ethics in the Use of Animals of the State University of Santa Catarina approved all experiments (protocol number 2322230522).

### 2.1. Experimental Design and Feeding

Twenty-four male Holstein calves with a maximum of five days of life were used and acquired through donations from regional producers. All calves had received adequate amounts of colostrum. When they arrived at the experiment site, they were housed in individual suspended cages measuring 100 cm × 120 cm × 107 cm, lined with rubber matting and wood shavings. The cages had support for water buckets and starters and support for individual nipple feeders. The calves were divided into control and phytobiotic groups, with 12 calves in each group, where each animal was considered an experimental unit. The animals were given a commercial milk substitute as a liquid diet, at a dose of 0.48 kg/day (in dry matter) diluted in two liters of water twice a day, at 08:00 and 16:30. For the animals in the phytobiotic group, a commercial mixture (Bezerrozan^®^, Tecphy, Chapecó, Brazil) of essential oils (cinnamon, oregano, and eucalyptus) was added, with a total purity of 6% of essential oils—that is, 60 mL/L in an emulsifying vehicle (glyceryl polyethylene glycol ricinoleate). The proportions of the three oils within the blend were the same (2%).

In the first 15 days of the experiment, a dose of 5 mL/feeding was given to each animal, and after that, the dose was increased to 10 mL/feeding. It was only given during lactation (60 days); the lowest initial dose was to adapt the animal’s taste to the product since it has a strong cinnamon smell. Both groups of animals received starter limited to a maximum intake of 2.5 kg/calf/day (natural matter) and water ad libitum. At the beginning of weaning (day 61), the amount of milk was proportionally reduced for 6 days, and the calves began to receive hay ad libitum in addition to the starter limited to 2.5 kg/day to avoid metabolic disturbances. The composition of the feed provided is displayed in Table 1.

### 2.2. Body Weight, Feed Efficiency and Stool Score

The feed was provided based on daily consumption, and the leftovers were weighed the following day to obtain the dry matter intake (DMI) per day. The animals were weighed on a digital scale weekly to monitor weight gain (WG: final weight—initial weight) and average daily gain (ADG: WG/number of days). Information feed efficiency (ADG/DMI—kg/kg) was obtained using mathematical calculations based on the animals’ weight and daily consumption. The stool score was evaluated daily using the visual analysis methodology proposed by the authors [27], in which the score varies from 1 to 5, where 1 is normal stool and 5 is entirely liquid stool due to the incidence of diarrhea. From score 3 onwards, diarrhea was considered, but with less severity.

### 2.3. Blood Analysis

Blood samples were collected for hematologic, biochemical, and immunological analyses on days 1, 15, 30, 45, 60, and 75. All the samples were collected in the morning, with the animals fasting from food and water. Collections were made through the jugular vein with needles and evacuated tubes. Tubes with EDTA were used to prevent clotting and enable hematologic analysis, and another contained a clot activator to obtain serum. For serum separation, the tubes with clot activator were centrifuged at 3500 RPM for 10 min, and the serum was transferred to identified microtubes and stored at –20 °C until further analyses were performed.

Hematologic analyses were performed using fresh blood on automatic equipment (Equipe Vet 3000^®^, Itatiba, Brazil). The equipment made it possible to quantify the total number of leukocytes and the differential (lymphocytes, granulocytes, and monocytes), total erythrocyte count, hemoglobin concentration, and hematocrit.

The biochemical analyses measured total protein, glucose, albumin, cholesterol, and urea serum levels on a semi-automatic analyzer (Bio-2000 BioPlus^®^, Barueri, Brazil) and commercial kits (Analisa^®^, Belo Horizonte, Brazil). The levels of globulins were obtained using a mathematical calculation (total proteins—albumin).

For protein fractionation, sodium dodecyl sulfate–polyacrylamide gel electrophoresis was performed, according to literature [28], adapted by other researchers [29] using a mini gel (10 × 10 cm). The gel was stained with Coomassie Blue and photographed to identify and quantify the protein fractions using Labimage1D software (https://www.kapelanbio.com/products/labimage/, accessed on 2 December 2024) Loccus Biotechnology, Morrisville, NC, USA). A standard containing fractions with molecular weight between 10 and 250 KD (Kaleidoscope—BIORAD, Hercules, CA, USA) was used as a reference.

Lipid peroxidation was determined through thiobarbituric-acid-reactive species (TBARS) levels using the methods described in literature [30] for sera samples. Serum TBARS were expressed as nM malondialdehyde (MDA)/mL. The measurement of total thiol levels was according to the methodology described by Ellman [31]. Glutathione S-transferase (GST) activity was determined according to the methodology described by Habig et al. [32].

### 2.4. Intestinal Microbiota Analysis

On days 1, 35, and 60, feces were collected directly from the rectal ampulla and stored in 3M™ Quick Swabs for qualitative and quantitative detection of microorganisms using metagenomics by sequencing the 16S rRNA gene, which was performed by the laboratory BPI—Biotechnology Research and Innovation^®^. It should be noted that animals undergoing antibiotic treatment due to diarrhea or other problems did not have their feces collected, as this would directly interfere with the analysis results.

Total DNA was extracted from 200 mg (wet weight) of samples with the ZR Fungal/Bacterial DNA MiniPrep kit (Zymo Research, Tustin, CA, USA). Primers 341F (5′-CCTAYGGGRBGCASCAG-3′) and 806R (5′-GGACTACNNGGGTATCTAAT-3′) were selected to amplify the V3–V4 region of bacterial 16S rRNA gene by polymerase chain reaction (PCR) [33].

Libraries were quantified by q-PCR using the Kapa Library Quantification Kit (Illumina, San Diego, CA, USA) following the manufacturer’s recommendations. Samples were normalized to a final concentration of 2 nM and sequenced with an Illumina MiSeq for 250 cycles from each end.

### 2.5. Statistical Analysis

A completely randomized design was used in this study. To analyze the data, they were first analyzed in a descriptive form. The data were then subjected to the normality test (Shapiro–Wilk test), which showed that all data had a normal distribution. We also evaluated the residual (including the command/influence residual after the fixed effects in the mixed model), which also showed normal distribution. Because of that, the data were analyzed using the SAS MIXED procedure (SAS Inst. Inc., Cary, NC, USA; version 9.4), with the Satterthwaite approximation being used to determine the denominator degrees of freedom for the fixed-effects test. Weight gain, average daily gain, feed conversion, and feed efficiency were tested for fixed treatment effects, and using the animal, as random variables. All other variables (body weight, biochemistry, blood count, protein count, and oxidants) were analyzed as repeated measures and tested for fixed effects of treatment, day, and treatment × day. All results obtained on d1 for each variable were also included as covariates; however, the command for covariates was removed from the model when *p* > 0.05. The Toeplitz covariance structure was selected for body weight (BW), and the first-order autoregressive covariance structure was selected for all other variables. The covariance structures were selected according to the lowest Akaike information criterion. Averages were determined using PDIFF, and all results were reported as LSMEANS followed by the standard error. The chi-square test was used for stool score analysis. Significant differences were defined when *p* ≤ 0.05 and trends when *p* > 0.05 and >0.10. We calculated the post-hoc power for the groups, considering feed efficiency. We used the mean of each group and its SEM with an alpha of 0.05, and we ended up with a reasonable power of 88.3%.

The sequence data were processed with Mothur v.1.39.5 [34], in line with the Mothur MiSeq SOP [35]. Taxonomy was then assigned by querying the representative sequence of each oligotype against the SILVA database (release 132) [36].

Closed-reference clustered OTU data were exported for analysis with Phyloseq v1.41 [37] in R 4.3.1 (R Core Team, 2023). The inverse Simpson and Shannon diversity index obtained alpha diversity values for bacterial communities. The inverse Simpson calculator is preferred to other alpha diversity measures since it indicates the richness of a community with uniform evenness that has the same level of diversity and thus has some biological interpretation [38]. According to literature, the inverse Simpson diversity combines richness with evenness [39], places more emphasis on common species [40], and is less sensitive to sampling efforts than the others alpha diversity indices [41]. PERMANOVA was applied to the differential test of distances among treatment groups [42]. The tax_glom function of Phyloseq was used to aggregate abundance data by genera to generate relative abundance plots of the 10 most abundant genera across all samples, plotted by averaging abundances by study and animal. Relative abundance of well-known potentially pathogenic *Clostridium* and *Escherichia* [43] was performed. All figures were generated with the ggplot2 package v3.2.1 in R [44]. 

## 3. Results

### 3.1. Clinical Changes

Some clinical interventions were necessary during the experiment, as described in Table S1. This happened due to problems such as diarrhea, pneumonia, and, in one case, a hoof injury. Diarrhea was the most observed clinical sign, and the use of antibiotics was only carried out following a protocol in which the animals presented a diarrhea score of 4 or 5 for more than 24 h, fever, apathy, and signs of dehydration. During this period, there was an infestation of flies (*Musca domestica*), which are very common in our region during hot periods (Guatambu/SC, Brazil, 27°09′07.0″ S 52°47′17.7″ W) as they can be vectors of various diseases. Stool score results are detailed in Figure S1.

### 3.2. Consumption, Weight Gain, and Feed Efficiency

Dry matter intake (DMI) tended to be lower in the phytobiotic group (*p* ≤ 0.1) than in the control group when we evaluated the total dry matter consumed throughout the experiment. The average initial weights of the calves were 40.7 kg and 41 kg, and the final weights were 78.1 kg and 79.6 kg, in the control and phytobiotic groups, respectively. The weight gains did not differ between groups in any of the measurements; however, animals in the phytobiotic group had better feed efficiency (*p* ≤ 0.05). The data are expressed in Table 2. A body weight curve for the animals was drawn up (Figure S2)

#### Stool Score

The treatment did not affect the stool score (*p* > 0.05—Figure S1). During the experiment, the score was 1.80 in the control group and 1.87 in the phytobiotic group. According to the methodology, scores of 1 and 2 were considered normal; however, it is worth noting that these values are averages of the entire experimental period, so we cannot say that the animals did not have diarrhea at any time.

### 3.3. Blood Tests

In the hematologic analysis, the animals in the phytobiotic group had lower leukocyte counts on days 45 and 60 because lymphocyte counts were also lower (*p* < 0.05). On day 30, the granulocyte count was also lower in the phytobiotic group (*p* < 0.05). Regarding the other hematologic parameters, no difference was observed during the experiment. The results are displayed in Table 3.

Glucose, total protein, albumin, urea, and globulin showed no difference throughout the experimental period. However, on days 30, 45, and 60, cholesterol levels were higher in the phytobiotic group (Table 4).

The results of the oxidative stress biomarkers are in Table 5. GST showed an effect of treatment and treatment × day interaction on days 15, 30, 45, and 60, higher in the serum of animals in the phytobiotic group. There was no treatment effect or treatment × day interaction for TBARS or total thiols.

The results of the proteinogram are displayed in Table 6, where we can observe an effect of treatment and treatment × day interaction for levels of IgA, ceruloplasmin, and transferrin. Serum IgA was higher in the phytobiotic group on day 60, ceruloplasmin was lower on days 30 and 60, and transferrin was lower on day 45. As an effect of treatment, this led to higher IgA and lower ceruloplasmin and serum transferrin in calves that consumed the blend of essential oils. There was no effect of treatment and treatment × day interaction for the concentration of heavy chain immunoglobulins, haptoglobin, C-reactive protein, and ferritin.

### 3.4. Intestinal Microbiota

**Figure 1:**
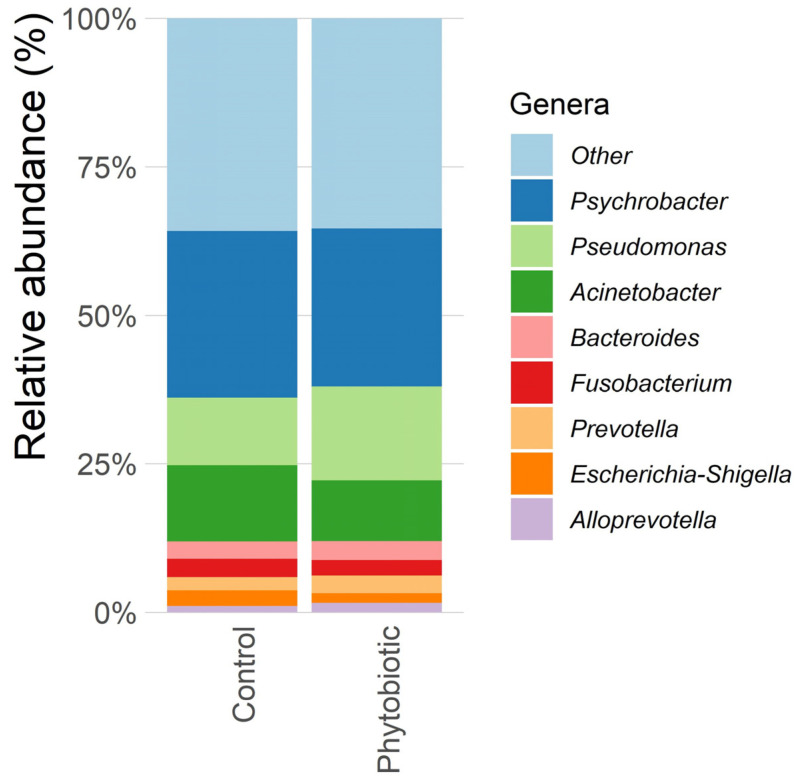
Ten most abundant bacterial genera found in the feces of calves supplemented with commercial mixture of essential oils. Abbreviations—Control: group of animals that did not receive the blend of essential oils; Phytobiotic: group of animals that received the blend. Data from the first collection (time 0) were excluded from this analysis.

**Figure 2:**
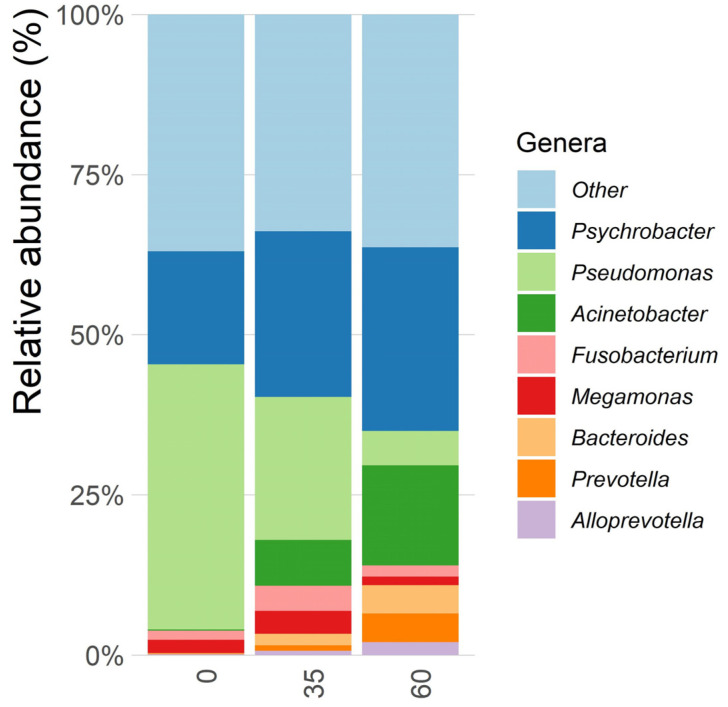
Ten most abundant bacterial genera found in calf feces on days 0, 35, and 60, dates that represent the days of the experiment.

From calf feces, nine genera of the most abundant microorganisms are shown in Figure 1 and Figure 2, detailed by treatments and experimental periods, respectively. There was a decrease in the relative abundances of the genera *Psychrobacter*, *Acinetobacter*, and *Escherichia* in phytobiotic group, compared with the control, but without statistical differences (*p* > 0.05). *Psychrobacter* is primarily a commensal that decomposes several dissolved organic carbon compounds, except for sugars, and its abundance increased along the experimental period [45]. *Acinetobacter* is considered an important human and veterinary pathogen, mostly due to intrinsic and acquired resistance to antimicrobials [46]. The designation “other” refers to genera that appeared in small amounts and genera that could not be identified against the SILVA database.

The relative abundances of phyla by groups in the feces of calves fed essential oils (phytobiotic) and control group (without essential oils) are presented in Figure S3. We highlight the phylum Proteobacteria, more abundant in the feces of calves, here.

**Figure 3:**
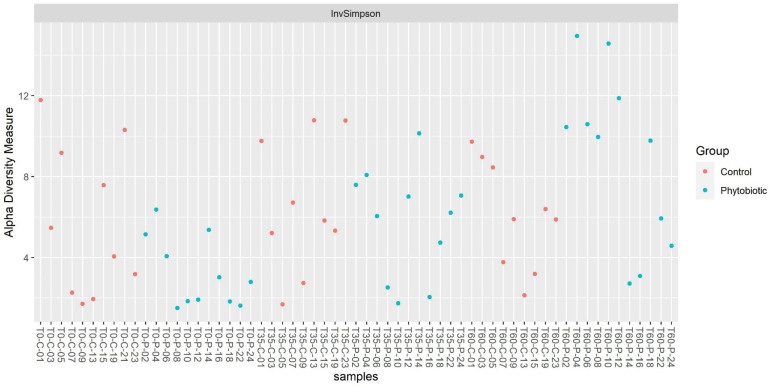
Alpha diversity of each sample. ‘Phytobiotic’ refers to the group that received the commercial mixture of essential oils.

**Figure 4:**
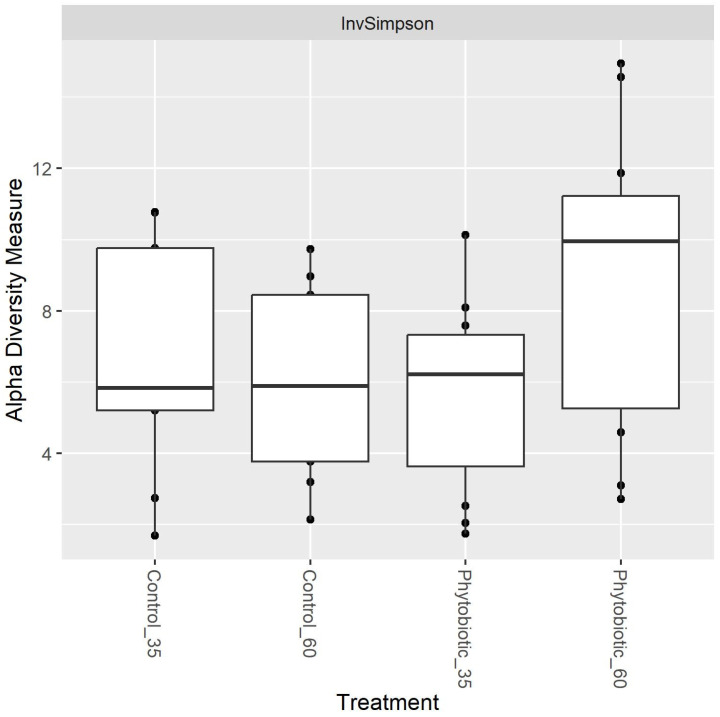
Alpha diversity based on treatment and days 35 and 60 of the experiment. ‘Phytobiotic’ refers to the group that received the blend of essential oils. Data from the first collection (time 0) were excluded from this analysis.

A more significant number of taxa were observed in the samples of animals from the phytobiotic group, demonstrating a greater alpha diversity than the control. Observing this alpha diversity, it is also possible to observe that the greatest diversity occurred later at the end of the experiment but without significant differences in treatment (*p* > 0.05) as shown in Figure 3 and Figure 4.

Alpha diversity considering the groups and the times of collection (d35 and d60) is presented in Figure S4. The difference within the phytobiotic group when comparing d35 with d60 when using the Shannon index (which was not observed in InvSimpon) stands out—that is, there was an increase in alpha diversity on day 60.

Beta diversity considering the groups and the collection times (d35 and d60) of calves that consumed essential oils is presented in Figure S5. PERMANOVA results were not significant (*p* > 0.05) for the treatments and no distinct clusters were found in the beta-diversity PCoA.

**Figure 5:**
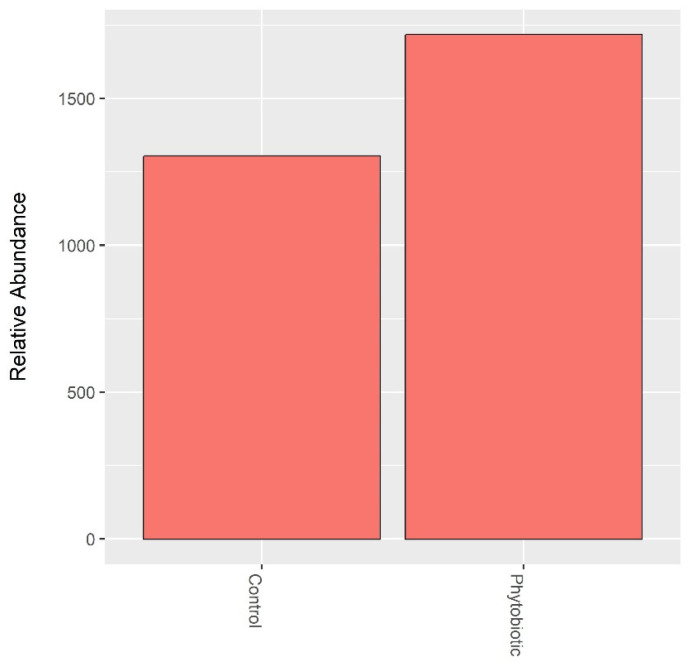
Abundance of *Clostridium* detected in calf feces. ‘Phytobiotic’ refers to the group that received the commercial essential oil blend.

**Figure 6:**
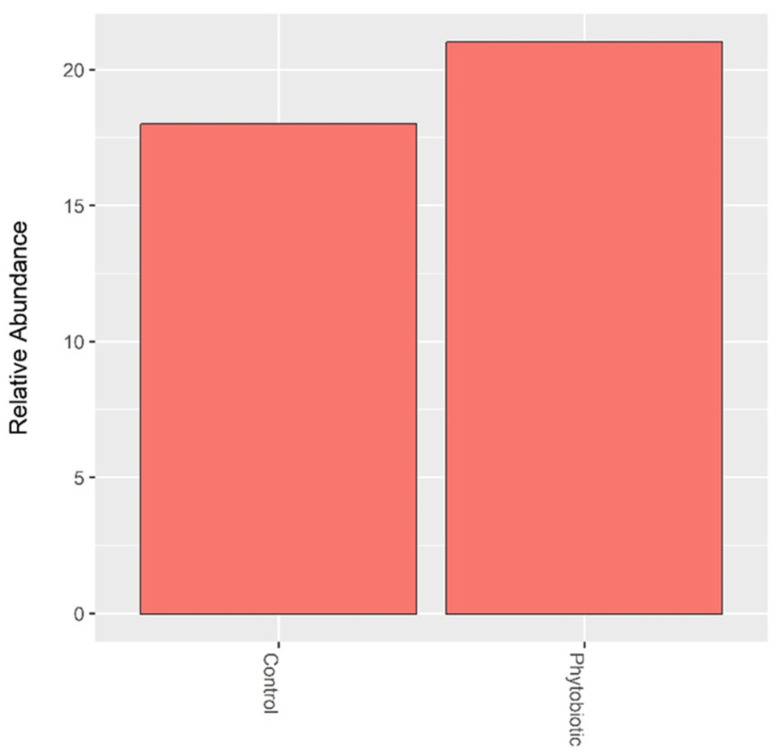
The abundance of *Escherichia* was detected in calf feces. ‘Phytobiotic’ refers to the group that received the commercial essential oil blend.

The abundance of *Clostridium* and *Escherichia* was assessed in isolation, as shown in Figure 5 and Figure 6. *Salmonella* was rarely identified. When comparing the treatments, there was no significant difference between the abundances of *Clostridium* and *Escherichia*.

## 4. Discussion

In general, the weight gain of the calves was lower than that found in most studies similar to ours described in this discussion. During the experimental period, some animals had an incidence of infectious diarrhea, and even pneumonia, which directly affected the performance of the calves, as there would be a loss of appetite resulting in a decrease in weight gain. Another critical point to be taken into consideration is the low amount of crude protein in the milk replacer. The recommendation is that it should be 20 to 22%, whether of dairy origin or not [47], and the milk replacer utilized contained 15%. However, despite this, consuming the blend of essential oils provided better feed efficiency for the calves because feed intake was lower, as researchers [48] had described when providing oregano oil to calves. Likewise, Silva et al. [49] obtained greater feed efficiency in lactating cows when supplied with a commercial additive composed of capsaicin, carvacrol, cinnamaldehyde, and eugenol, with carvacrol and cinnamaldehyde being two of the components present as bioactives in the product used in the experiment in question.

Furthermore, Farshid et al. [50] found greater feed efficiency for calves supplemented with cinnamon essential oil than those supplemented with oil from other plants and a probiotic. The more significant weight gain with lower DMI can be explained by a slight modulation of ruminal microorganisms, allowing greater efficiency in using available nutrients. Essential oils can alter rumen fermentation, altering the ratio of volatile fatty acids from food degradation and even protein metabolism, becoming a performance improver precisely for this reason [13,51], favoring the animal nutritionally. Therefore, our results corroborate the literature, showing that this tested essential oil blend is a way to save money on feed costs for raising calves without losing productivity.

Although there was no statistical difference in weight gain and average daily gain, numerically, the phytobiotic group had lower rates, which was different from what was expected in studies with essential oils, where there was a higher performance rate. It is believed that this occurred due to sanitary conditions; in Table S1, it is possible to observe the incidence of diseases, mainly infectious diarrhea, which is known to affect the calf’s consumption and weight gain directly, and the incidence of diarrhea occurred more in animals in the phytobiotic group than the control group. Nevertheless, it was still not harmful in any significant way.

Another point that drew attention was that cholesterol was higher in the phytobiotic group despite the lower consumption of starters. Serum cholesterol is the balance of cholesterol that enters the bloodstream, endogenous or exogenous, with the output of the same metabolism [52]. Furthermore, years ago, as in the work of the authors of [53], it was proven that the fat quality in the diet affects cholesterol concentrations more than the quantity of fat ingested.

The cholesterol level in some studies was shown to be inverse to the consumption of a starter, regardless of the composition of the substitute or the addition of essential oils, as shown in our study. In one study by researchers [54], calves were fed with the same essential oils as in the present study, oregano, eucalyptus, and cinnamon, with higher cholesterol levels in the group where starter consumption was lower. Furthermore, in the survey by Wilms [55], where substitutes with different fat sources were provided, the highest cholesterol levels were in calves fed with the substitute containing fat of vegetable origin, the most similar to that used in our study, and this group was also the one with the lowest starter consumption. As previously mentioned, the presence of high or low cholesterol indicates that metabolism is out of balance, being affected by the quality of the fat [52,53]. With this in mind, we believe that the substitute supplied had fat of a lower quality than the fat in the concentrate, reducing this metabolism and increasing serum cholesterol since the consumption of higher-quality fat was reduced.

The components of the essential oils in this work are considered to be antimicrobials and intestinal flora modulators, and one of the possible responses to this antimicrobial action is a decrease in the incidence of diarrhea. Previous researchers [48,56] gave oregano oil to calves and obtained a lower incidence of diarrhea; there was less use of antibiotics in a group of animals that were consuming essential oil [56]. Ritt et al. [57] concluded that an oregano extract with 80% carvacrol positively affected the intestinal and ruminal bacterial population, decreasing the abundance of the pathogenic bacteria *Streptococcus*, *Clostridium*, and *Escherichia*, unlike in the present study, where no difference was obtained for *Clostridium* and *Escherichia* between treatments. The difference between the doses offered may explain this since, in the present study, the amount used was low compared with the literature values. The proportion of the three essential oils was the same, which could also have been a problem since oregano essential oil is the most cited antimicrobial, and a higher proportion of it would make a significant difference to the antimicrobial effects of the primary pathogens that cause diarrhea.

Cinnamon, oregano, and eucalyptus oils also improve immune and antioxidant systems. Like in our study, the authors of [58] obtained a better immune response, with higher values of IgM, IgG, and IgA, when feeding calves with an essential oil also containing probiotics (carvacrol, caryophyllene, p-cymide, cineol, terpinene, and thymol). Al-Suwaiegh et al. [59] achieved higher levels of total protein and globulin, in addition to positive results in cows’ milk production with the supply of a mixture of clove, oregano, and juniper oils. IgA is an antibody predominantly produced on mucosal surfaces and indicates intestinal immunity [60] because it coats pathogenic microorganisms, protecting the host against infection [61]. However, the function of IgA is to act on the integrity of the intestinal barrier, enhancing pro-inflammatory immune responses and effectively contributing to intestinal homeostasis [60]. This fact demonstrates that the animals in the phytobiotic group had greater intestinal protection, probably due to the blend of oils, avoiding the incidence of diarrhea and diseases caused by pathogens and enhancing the immune responses.

Acute-phase proteins such as ceruloplasmin, haptoglobin, and transferrin are produced by the liver [62,63]; they were detected in lower concentrations in the phytobiotic group at some times during the experimental period. The response of these proteins is a nonspecific reaction that occurs shortly after the damage caused by the pathogen [63]. Furthermore, their concentrations indicate the intensity of the inflammatory response in the body [63], which may have been lower in the phytobiotic group in the present study due to the known anti-inflammatory effects of essential oils. Erkiliç et al. [64] showed that neonatal calves that show clinical signs of diarrhea, the absence of a sucking reflex, dehydration, cold extremities, lateral recumbency, and an inability to stand have higher serum levels of ceruloplasmin, showing that high levels indicate that the animal is not is in good health. Saleh et al. [65] showed that calves with diarrhea had increased levels of ceruloplasmin (which was expected) as it is an inflammatory response. For the present study, we believe in an anti-inflammatory effect caused by the blend of essential oils since transferrin and ceruloplasmin are markers of inflammatory intensity. Higher levels of IgA [66] lower levels of leukocytes [67], and lower acute-phase proteins [68] in the phytobiotic group indicated that the calves had improved immune systems and overall health; however, we cannot rule out other mechanisms involved and related to the changes.

Lower levels of leukocytes and lymphocytes have been detected in lambs supplemented with essential oil containing carvacrol, thymol, and cinnamaldehyde [69], as in the present experiment. This finding can be explained by the antimicrobial action of oils [69] because oils change the permeability of the bacteria membrane [70]. The action of oils minimizes energy expenditure during inflammatory responses and directs this energy toward growth [71], associated with better feed efficiency. The blend of essential oils provided the animals with antioxidant mechanisms since the lipid peroxidation in the phytobiotic group was lower than that of the control group. This finding was due to a higher activity of GST, which inhibits oxidative reactions [72].

The nine bacterial genera detected in the feces of calves in our experiment, both control and treatment, were *Acinetobacter*, *Alloprevotella*, *Bacteroides*, *Collinsella*, *Fusobacterium*, *Megamonas*, *Prevotella*, *Pseudomonas*, and *Psychrobacter*; among them, *Acinetobacter*, *Pseudomonas*, and *Psychrobacter* were the most abundant. *Acinetobacter* has animals as one of its natural habitats as it has high resistance to antimicrobials and the environment, with the species of *A. baumannii* being the most significant of its kind in animals [73]. This bacterium can cause infections of different types, including in soft tissues such as the intestine, also causing symptoms of pneumonia [74], a common disease in calves. In the distribution of calves by experimental period, *Acinetobacter* levels increased significantly on days 35 and 60, which can be explained by the fact that it belongs to the normal intestinal microbiota in calves, and its growth occurs as the microbiota is established.

*Pseudomonas*, by contrast, lives in different environments using animal tissues as a substrate, also having substantial resistance to antimicrobials [75,76,77,78]. It is a bacterium associated with meat deterioration and rarely associated with animal health [77,78]. *P. aeruginosa* is the most important in animals because it is not a constituent of the normal intestinal microbiota [76,77], and the lower abundance of this genus throughout the day can be considered beneficial for the health of our calves. *Psychrobacter* is also an opportunistic pathogen [79]. Rama et al. [80] identified *Psychrobacter* as one of the most abundant genera in the intestinal microbiota of healthy broilers, whether in conventional systems or systems without antibiotics. As they are opportunistic, the presence of these three genera, as already mentioned, will only cause damage to the animal’s health when immunosuppressed.

When we analyze the distribution of the nine genera within each experimental period (days 0, 35, and 60), we notice a fluctuation in the bacterial population as the animal aged and the microorganisms became established. In addition to *Acinetobacter*, the genus *Bacteroides* was practically non-existent on day zero but was evident throughout the experimental period. However, more emphasis was needed when evaluating only the treatment. This genus is also part of the normal intestinal microbiota. However, *B. fragilis* is clinically significant [77,81] as it produces an enterotoxin that causes diarrhea in calves [82]. The pathogenic potential of *B. fragilis* is similar to that of *E. coli* [83], which is one of the main microorganisms that causes diarrhea in calves and is related to a high mortality rate [81]. However, many *Bacteroides* sp. produce enzymes such as superoxide dismutase, which acts directly on the antioxidant system [82], beneficial to the animal’s body when it is not immunosuppressed.

Another genus found was *Fusobacterium*, which remained regular during the experimental period and without difference between groups. *F. necrophorum* is one of the most prevalent Fusobacteria, a typical gastrointestinal microorganism [84]. The genus *Megamonas* can be associated with healthy individuals [85] and is related to producing beneficial substances and reducing pathogenic bacteria abundance [20,86].

The genus *Alloprevotella* became evident only after 60 days of the experiment, being a group of beneficial bacteria since they can produce short-chain fatty acids [87], allowing them to be associated with improved performance of the animal [88]. The literature suggests that the *Alloprevotella* genus acts as an intestinal barrier stimulator, reducing the numbers of pathogenic organisms through competition and the need for antibiotics [89,90]. According to the literature, the genera *Prevotella*, *Bacteroides*, and *Fusobacterium* can be found in greater abundance from the 15th day of life in calves, when solid intake is increasing [91], which explains the greater presence of these genera in feces collected on days 35 and 60.

No difference was observed between treatments in the population abundances of *E. coli* and *Clostridium* spp. *E. coli* is the primary bacterium causing calf diarrhea [92,93], but its presence does not mean that the animal will be sick since its pathogenicity depends on two virulence factors that need to be present [92]; therefore, the bacteria is a constituent of the normal microbiota. *Clostridium* is another bacteria responsible for causing diarrhea in calves, primarily *C. perfringens*; however, it is less common and is found in the microbiota in healthy animals [93]. The presence of these two bacteria throughout the experiment did not significantly harm health as they were immunologically protected, as observed in the results of immunological biomarkers such as the number of leukocytes and immunoglobulin concentration. Furthermore, the mean fecal scores were 1.80 and 1.87 in the control and phytobiotic groups, respectively, and Figure S2 shows the distribution of the fecal score every two weeks. Even with incidences of diarrhea that required clinical interventions, the fecal score values were low since 1 and 2 indicate normal feces, which shows that, in general, the animals demonstrated adequate intestinal conditions most of the time.

Diarrhea in calves is very economically damaging to the dairy industry as calves will require treatment with antimicrobials and other drugs. These animals will be sensitive throughout their lives, which can lead to reproductive problems and low milk production depending on the degree and frequency of diarrhea [94]. In addition, diseases associated with diarrhea in calves are responsible for causing more than 50% mortality due to the other clinical signs that occur together [95]. Although the essential oils studied in this study have proven antimicrobial properties, this was not observed in our study. It is believed that this occurred because the animals were in a controlled environment, with proper and routine handling and sanitization, which may have had an impact on these results, since one hypothesis is that if the animals were in a sanitary challenge, the antimicrobial effects of the essential oils could have been observed as possible modulators of the intestinal microbiota, reducing the bacterial load of the microorganisms that cause diarrhea.

## 5. Conclusions

The conclusion of this study must be made with caution because the results indicated that the supply of a blend of essential oils with cinnamon, oregano, and eucalyptus provided the calves with humoral and antioxidant immune system stimulation, minimizing physiological oxidative stress and better efficiency. However, this health improvement did not enhance the growth of calves that consumed essential oils. The assessments of the abundance and biodiversity of the microbiota did not differ between animals that consumed the blend of essential oils and those that did not; however, it allowed the identification of the main genera in the feces of calves in the first weeks of age. Despite this, in this study, the essential oils did not play an antimicrobial role, in line with the reports already found in the literature.

## Figures and Tables

**Table 1:** Ingredients and chemical composition of feed fed to calves.

Ingredients		Quantity (Dry Matter) Days 1–60	Quantity (Dry Matter) Days 61–75
STEP 1			
Starter ^1^		Ad libitum	Ad libitum
Milk replacer		484 g/day	363 g/day (days 61–62), 242 g/day (days 63–64); and 121 g/day (days 65–66)
Hay		0	Ad libitum
Chemical composition in dry matter (g/kg)			
	Starter	Replacer	Hay
Dry matter	893	973	850
Crude protein	261	279	72.3
Ether extract	34.4	92.5	8.00
Ash	64.4	78.5	72.3
Crude fiber	-	14.0	-
NDF	260	-	602
ADF	94.8	-	242
Lactose	-	536	-

^1^ The starter was based on wheat bran, micronized soy bran, ground corn, whey powder, and mineral and vitamin supplement. The starter had the following minimum guaranteed levels: Calcium (9–11.00 g/kg), phosphorus (6800 mg/kg), sodium (1900 mg/kg), magnesium (1500 mg/kg), sulfur (2800 mg/kg), cobalt (5 mg/kg), copper (40 mg/kg), iron (68 mg/kg), iodine (4.40 mg/kg), manganese (80 mg/kg), selenium (1 mg/kg), zinc (96 mg/kg), vitamin A (7500 IU/kg), vitamin D3 (1500 IU/kg), vitamin E (7.5 IU/kg), vitamin K3 (1.2 mg/kg), vitamin B1 (0.60 mg/kg), vitamin B2 (2 mg/kg), vitamin B6 (1.50 mg/kg), vitamin B12 (11 mcg/kg), vitamin B3 (15 mg/kg), vitamin B9 (0.30 mg/kg), vitamin C (0.35 mg/kg), choline (210 mg/kg), sodium monensin (36 mg/kg), and flavomycin (0.72 mg/kg).

**Table 2:** Growth performance and consumption of calves in the phytobiotic group fed with a commercial blend of essential oils, and also in the control group.

Variable	Control	Phytobiotic	SEM	*p*—Treat ^1^	*p*—Treat × Day ^2^
Body weight, kg				0.86	0.87
d1	40.7	41.0	0.34		
d60	61.2	62.4	0.34		
d75	78.1	79.6	0.35		
WG, kg					
d1–60	16.6	16.0	0.21	0.84	–
d1–75	34.4	36.0	0.22	0.39	–
d60–75	17.6	20.0	0.20	0.42	–
ADG, kg					
d1–60	0.27	0.26	0.03	0.88	–
d60–75	1.24	1.33	0.04	0.80	–
DMI of starter, kg DM					
d1–60	0.52	0.41	0.05	0.11	–
d60–75	1.74	1.52	0.16	0.11	–
DMI of milk replacer, kg DM					
d1–60	0.484	0.484	0.00	–	–
DMI: starter + milk replacer, kg DM					
d1–60	1.00 ^a^	0.90 ^b^	0.04	0.08	–
Feed efficiency, kg/kg					
d1–60	0.27 ^b^	0.29 ^a^	0.03	0.05	–

Phytobiotic: treatment group, which received the blend of essential oils. ^1^ The table shows treatment effect, where different letters on the same line show significant differences (*p* ≤ 0.05) and trends (*p* > 0.05 and ≤0.1) between groups. ^2^ It also shows treatment × day interaction, where different letters on the same line show significant differences (*p* ≤ 0.05) and trends (*p* > 0.05 and ≤0.1) between groups. Abbreviations: dry matter (DM), weight gain (WG), average daily gain (ADG), and dry matter intake (DMI).

**Table 3:** Blood count of calves in the phytobiotic group fed with a commercial essential oil blend, and also in the control group.

Variable	Control	Phytobiotic	SEM	*p*—Treat ^1^	*p*—Treat × Day ^2^
Leukocytes (×10^3^ cel/µL)				0.25	0.01
d1	14.8	16.6	0.69		
d15	16.6	15.8	0.66		
d30	21.2	17.3	0.77		
d45	12.4 ^a^	9.91 ^b^	0.65		
d60	8.00 ^a^	5.17 ^b^	0.66		
d75	8.24	7.53	0.66		
Lymphocytes (×10^3^ cel/µL)				0.07	0.01
d1	8.22	7.87	0.54		
d15	10.8	8.58	0.54		
d30	10.9	10.5	0.54		
d45	8.53 ^a^	5.60 ^b^	0.52		
d60	6.70 ^a^	3.74 ^b^	0.52		
d75	4.62	3.96	0.52		
Monocytes (×10^3^ cel/µL)	1.78	1.38	0.61	0.52	0.21
Granulocytes (×10^3^ cel/µL)				0.75	0.05
d1	3.78	4.91	0.35		
d15	3.32	4.37	0.35		
d30	6.57 ^a^	4.72 ^b^	0.35		
d45	2.02	2.66	0.34		
d60	2.07	2.35	0.29		
d75	2.38	2.32	0.28		
Erythrocytes (×10^6^ cel/µL)	8.05	8.31	0.03	0.95	0.96
Hemoglobin (mg/dL)	10.4	10.4	0.18	0.98	0.95
Hematocrit (%)	28.4	28.3	0.71	0.96	0.94

Phytobiotic: treatment group, which received the blend of essential oils. ^1^ The table shows treatment effect, where different letters on the same line show significant differences (*p* ≤ 0.05) and trends (*p* > 0.05 and ≤0.1) between groups. ^2^ The table also shows treatment × day interaction, where different letters on the same line show significant differences (*p* ≤ 0.05) and trends (*p* > 0.05 and ≤0.1) between groups.

**Table 4:** Clinical biochemistry of calves fed with commercial mixture of essential oils.

Variables	Control	Phytobiotic	SEM	*p*—Treat ^1^	*p*—Treat × Day ^2^
Glucose (mg/dL)	83.5	83.1	2.12	0.95	0.98
Cholesterol (mg/dL)				0.05	0.01
d1	59.5	59.8	4.52		
d15	75.8	82.4	4.61		
d30	144 ^b^	161 ^a^	4.27		
d45	109 ^b^	150 ^a^	5.81		
d60	112 ^b^	147 ^a^	5.92		
d75	120	131	4.48		
Total protein (g/dL)	6.52	6.34	0.25	0.56	0.30
Albumin (g/dL)	2.64	2.65	0.09	0.95	0.92
Urea (mg/dL)	17.2	16.8	0.55	0.91	0.88
Globulin (g/dL)	3.87	3.68	0.18	0.81	0.76

Phytobiotic: treatment group, which received the blend of essential oils. ^1^ The table shows treatment effect, where different letters on the same line show significant differences (*p* ≤ 0.05) and trends (*p* > 0.05 and ≤0.1) between groups. ^2^ The table also shows treatment × day interaction, where different letters on the same line show significant differences (*p* ≤ 0.05) and trends (*p* > 0.05 and ≤0.1) between groups.

**Table 5:** Oxidative status of calves fed with commercial mixture of essential oils.

Variables	Control	Phytobiotic	SEM	*p*—Treat ^1^	*p*—Treat × Day ^2^
TBARS (g/dL)	17.3	16.3	1.02	0.81	0.86
GST (g/dL)				0.01	0.01
d1	432	414	5.84		
d15	420 ^b^	457 ^a^	5.85		
d30	411 ^b^	525 ^a^	5.74		
d45	410 ^b^	492 ^a^	5.79		
d60	420 ^b^	539 ^a^	5.78		
Total Thiols (g/dL)	62.2	65.7	1.03	0.76	0.62

Phytobiotic: treatment group, which received the blend of essential oils. TBARS: thiobarbituric-acid-reactive substances; GST: glutathione S-transferase enzyme. ^1^ The table shows treatment effect, where different letters on the same line show significant differences (*p* ≤ 0.05) and trends (*p* > 0.05 and ≤0.1) between groups. ^2^ The table also shows treatment × day interaction, where different letters on the same line show significant differences (*p* ≤ 0.05) and trends (*p* > 0.05 and ≤0.1) between groups.

**Table 6:** Proteinogram of calves fed or not with commercial mixture of essential oils.

Variables	Control	Phytobiotic	SEM	*p*—Treat ^1^	*p*—Treat × Day ^2^
IgA (g/dL)				0.05	0.03
d1	0.69	0.65	0.02		
d15	0.67	0.64	0.02		
d30	0.70	0.71	0.02		
d45	0.65	0.70	0.03		
d60	0.62 ^b^	0.75 ^a^	0.02		
Heavy chain Ig (g/dL)	0.96	1.01	0.04	0.38	0.17
Ceruloplasmin (g/dL)				0.05	0.02
d1	0.52	0.54	0.02		
d15	0.53	0.50	0.02		
d30	0.58 ^a^	0.51 ^b^	0.01		
d45	0.52	0.48	0.01		
d60	0.50 ^a^	0.39 ^b^	0.02		
Haptoglobin (g/dL)	0.29	0.30	0.01	0.89	0.60
C-reactive protein (g/dL)	0.18	0.16	0.01	0.45	0.23
Ferritin (g/dL)	0.23	0.20	0.02	0.54	0.42
Transferrin (mg/dL)				0.01	0.01
d1	0.20	0.20	0.02		
d15	0.21	0.20	0.02		
d30	0.20	0.17	0.02		
d45	0.21 ^a^	0.16 ^b^	0.01		
d60	0.22	0.17	0.02		

Phytobiotic: treatment group, which received the blend of essential oils. ^1^ The table shows treatment effect, where different letters on the same line show significant differences (*p* ≤ 0.05) and trends (*p* > 0.05 and ≤0.1) between groups. ^2^ The table also shows treatment × day interaction, where different letters on the same line show significant differences (*p* ≤ 0.05) and trends (*p* > 0.05 and ≤0.1) between groups.

## Data Availability

Data remain with the authors but may be made available upon request.

## Supplementary Materials

The following supporting information can be downloaded at: https://www.mdpi.com/article/10.3390/ani14243555/s1, Table S1: Clinical interventions during the experiment; Figure S1: Fecal score of calves fed essential oils blend diluted in milk (d1–60) and follow-up after weaning (d61–75); Figure S2: Illustration of the evolution of calves’ body weight; Figure S3: Relative abundance of phyla by groups in the feces of calves fed essential oils (phytobiotic) and control group (without essential oils); Figure S4: Alpha diversity considering the groups and the collection time (d35 and d60) of calves that consumed essential oils (phytobiotic group) compared to the control (without additive). Statistical difference (*p* < 0.05) was illustrated by different letters (a,b) in the graph, above the bar; Figure S5: Beta-diversity considering the groups and the collection time (d35 and d60) of calves that consumed essential oils (phytobiotic group) compared to the control (without additive).
