# Supplementary material for: The Effects of a Blend of Essential Oils in the Milk of Suckling Calves on Performance, Immune and Antioxidant Systems, and Intestinal Microbiota
Source: Animals (Basel). 2024 Dec 10;14(24):3555. doi: 10.3390/ani14243555 (PMC11672722; doi:10.3390/ani14243555)

Table S1. Clinical interventions during the experiment.

ANIMALS	GROUP	INTERVENTION	REASON	DIARRHEA FREQUENCY	DIARRHEA DUARTION (days)
6	Phytobiotic	Gentamicin sulfate	Infectious diarrhea score 5	1	2 (d6 – d7)
8	Phytobiotic	Gentamicin sulfate	Infectious diarrhea score 5	1	3 (d12 – d14)
		Enrofloxacina + Piroxicam	Infectious diarrhea score 5	1	2 (d48 – d49)
9	Control	Gentamicin sulfate	Infectious diarrhea score	1	4 (d6 – d9)
		Enrofloxacina + Piroxicam	Pneumonia	Does not apply	Does not apply
10	Phytobiotic	Gentamicin sulfate	Infectious diarrhea score 5	2	5 (d2 – d6) e 2 (d39 – d40)
		Enrofloxacina + Piroxicam	Infectious diarrhea score 5	1	3 (d29 – d31)
		Ampicillin + colistin + dexamethasone	Infectious diarrhea score 5	1	2 (d48 – d49)
11	Control	Gentamicin sulfate	Infectious diarrhea score 5	1	4 (d30 – d33)
		Enrofloxacina + Piroxicam	Infectious diarrhea score 5	1	2 (d29 – d40)
		Ceftiofur hydrochloride	Infectious diarrhea score 5	1	6 (d53 – d58)
		Meloxicam + dipyrone sodium	Hoof injury	Does not apply	Does not apply
12	Phytobiotic	Gentamicin sulfate	Infectious diarrhea score 5	2	2 (d6 – d7) e 5 (d46 – d50)
		Enrofloxacina + Piroxicam	Infectious diarrhea score 5	1	2 (d23 – d25)
		Ceftiofur hydrochloride	Infectious diarrhea score 5	1	4 (d41 – d44)

14	Phytobiotic	Gentamicin sulfate	Infectious diarrhea score 5	1	3 (d49 – d51)
15	Control	Gentamicin sulfate	Infectious diarrhea score 5	1	2 (d11 – d12)
16	Phytobiotic	Gentamicin sulfate	Infectious diarrhea score 5	1	2 (d14 – d15)
		Enrofloxacina + Piroxicam	Infectious diarrhea score 5	1	5 (d38 – d42)
		Ceftiofur hydrochloride	Infectious diarrhea score 5	1	4 (d49 – d52)
17	Control	Enrofloxacina + Piroxicam	Infectious diarrhea score 5	1	3 (d39 – d41)
18	Phytobiotic	Gentamicin sulfate	Infectious diarrhea score 5	1	2 (d4 – d5)
		Enrofloxacina + Piroxicam	Infectious diarrhea score 5	1	3 (d24 – d26)
20	Phytobiotic	Enrofloxacina + Piroxicam	Infectious diarrhea score 5	2	3 (d37 – d39) e 2 (d61 – d62)
		Gentamicin sulfate	Infectious diarrhea score 5	1	2 (d3 – d4)
		Ampicillin + colistin + dexamethasone	Infectious diarrhea score 5	1	6 (d40-d45)
21	Control	Gentamicin sulfate	Infectious diarrhea score 5	1	2 (d6 – d7)
22	Phytobiotic	Gentamicin sulfate	Infectious diarrhea score 5	1	2 (d2 – d3)
		Enrofloxacina + Piroxicam	Infectious diarrhea score 5	1	3 (d14 – d16)

*There are 24 experimental units, but only calves that received clinical interventions are described in this table.

Figure S1. Fecal score of calves fed essential oils blend diluted in milk (d1-60) and follow-up after weaning (d61-75).

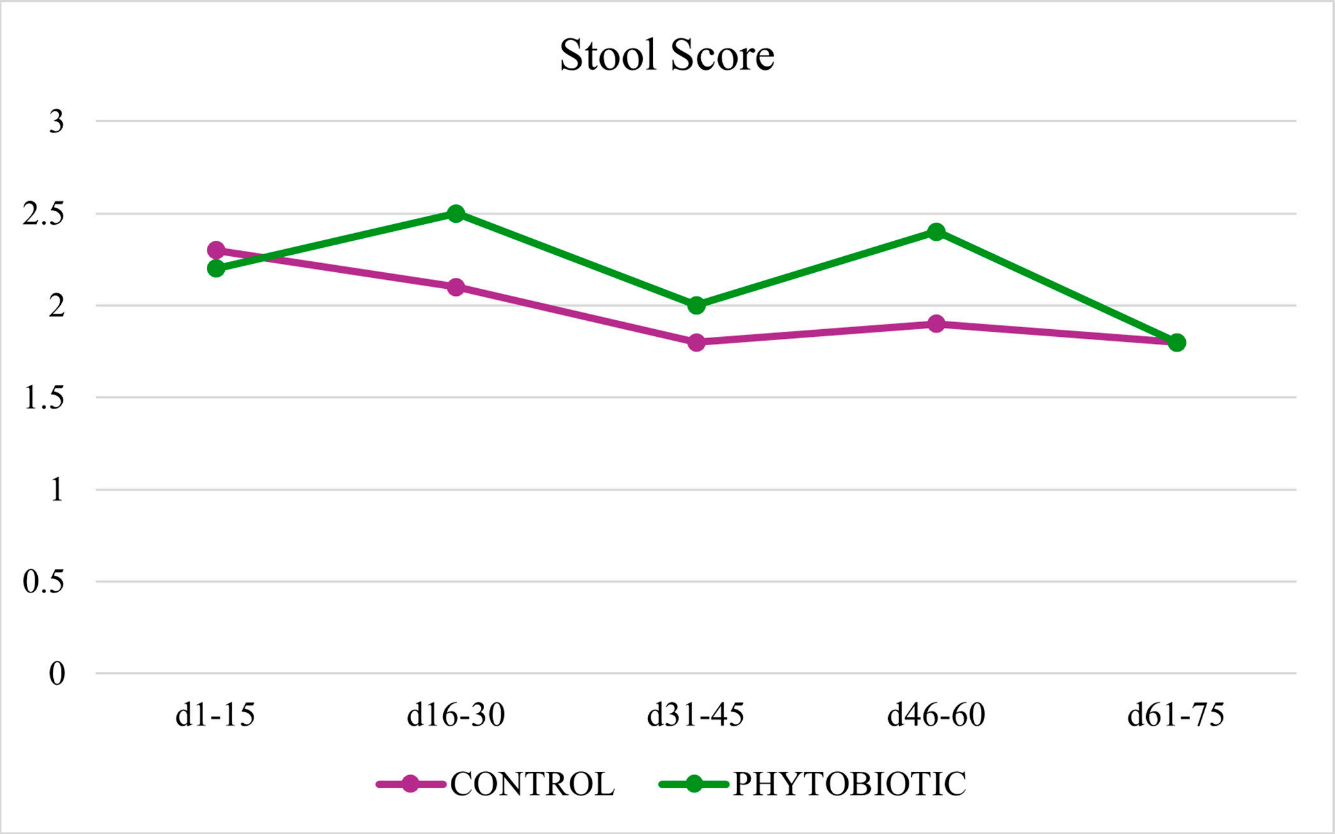

Figure S2. Illustration of the evolution of calves' body weight.

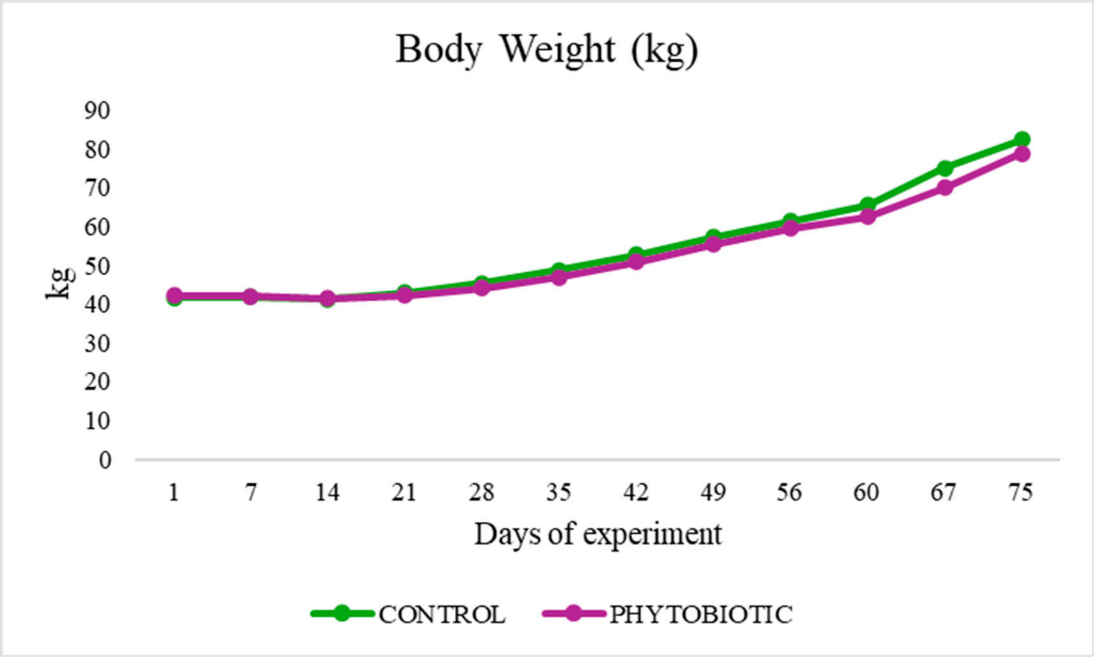

Figure S3: Relative abundance of phyla by groups in the feces of calves fed essential oils (phytobiotic) and control group (without essential oils).

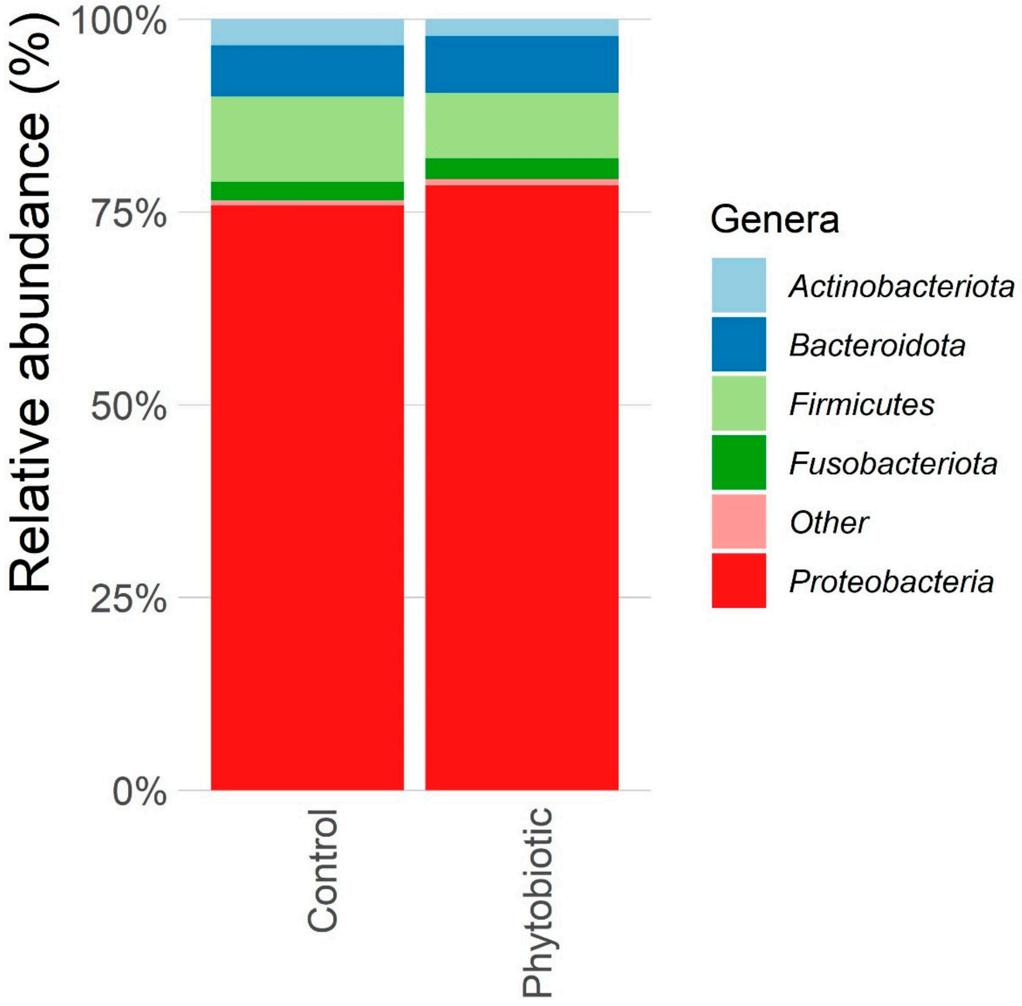

Figure S4: Alpha diversity considering the groups and the collection time (d35 and d60) of calves that consumed essential oils (phytobiotic group) compared to the control (without additive). Statistical difference (P < 0.05) was illustrated by different letters (a,b) in the graph, above the bar.

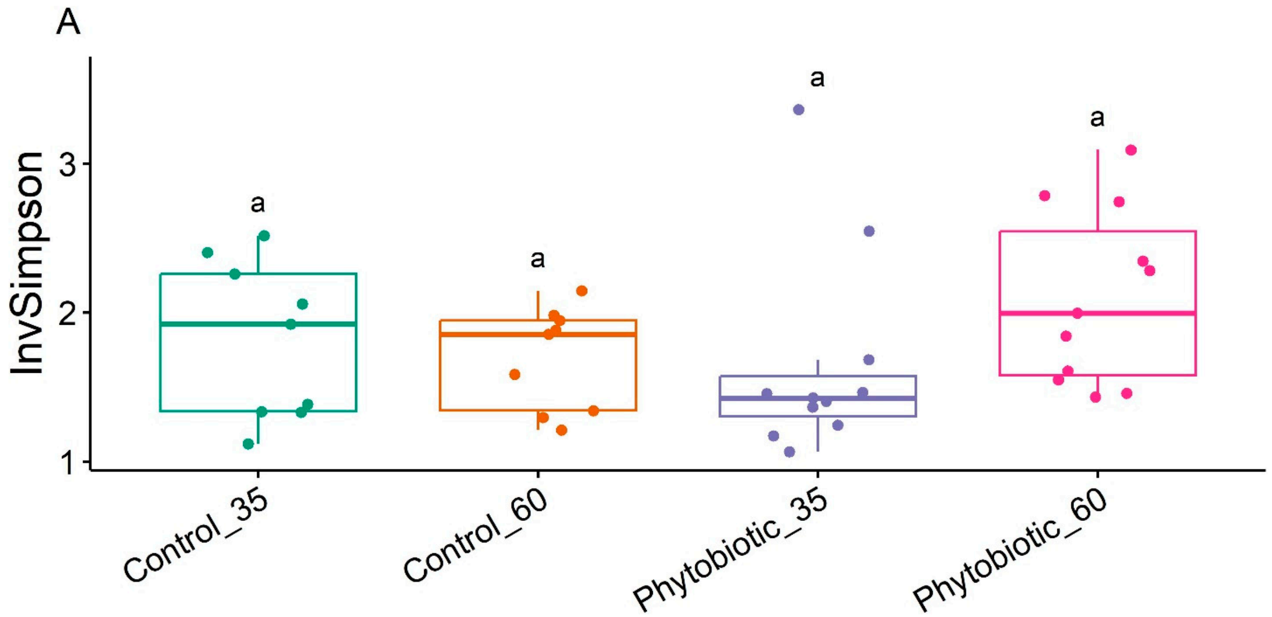

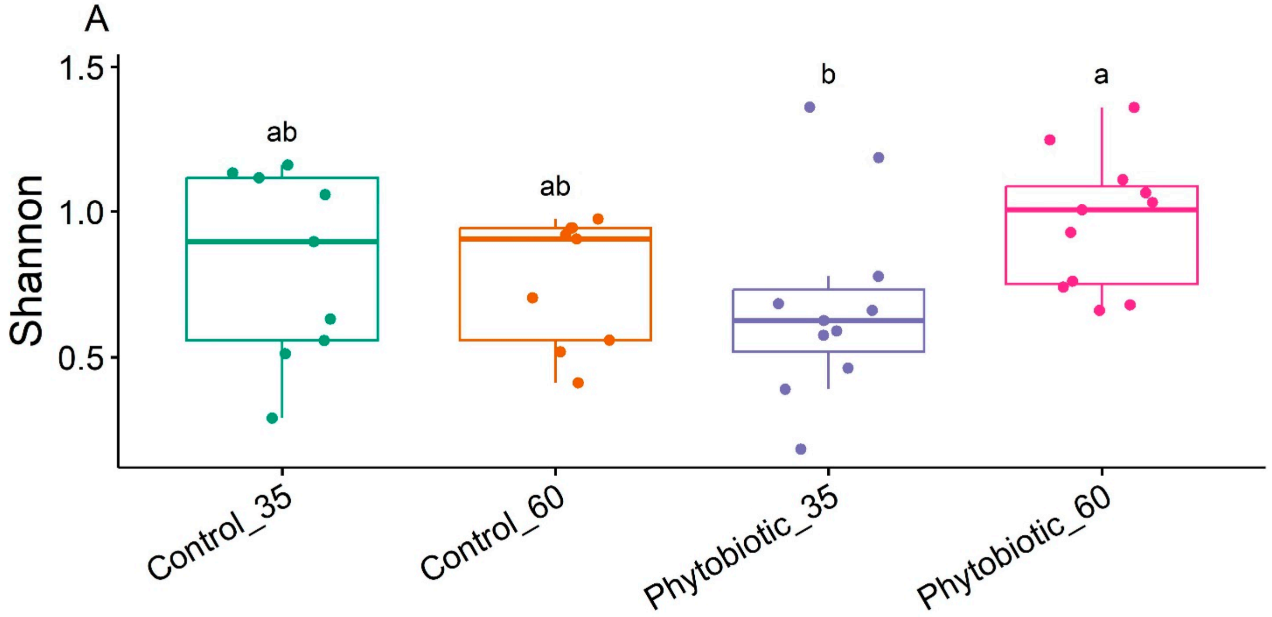

Figure S5. Beta-diversity considering the groups and the collection time (d35 and d60) of calves that consumed essential oils (phytobiotic group) compared to the control (without additive).

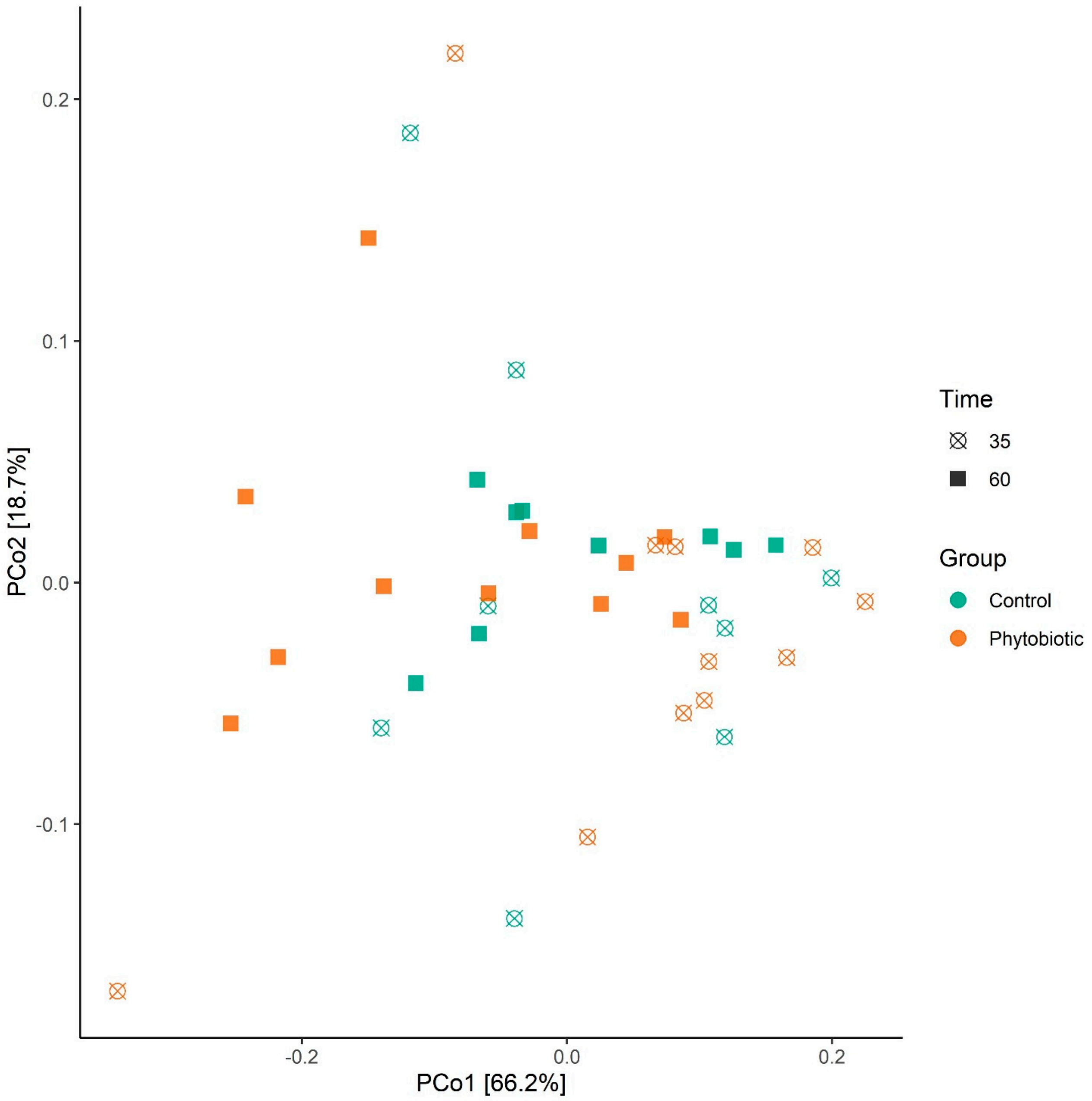

Supplement: Supplementary file 1 [file animals-14-03555-s001.zip › animals-3316645-supplementary.pdf]
